# Valve-sparing David procedure *via* minimally invasive access does not compromise outcome

**DOI:** 10.3389/fcvm.2022.966126

**Published:** 2022-10-14

**Authors:** Malakh Shrestha, Tim Kaufeld, Pamila Shrestha, Andreas Martens, Saad Rustum, Linda Rudolph, Heike Krüger, Morsi Arar, Axel Haverich, Erik Beckmann

**Affiliations:** Department of Cardiothoracic, Transplantation and Vascular Surgery, Hannover Medical School, Hanover, Germany

**Keywords:** aortic valve-sparing root replacement, David procedure, reimplantation procedure, minimally invasive surgery, mini access

## Abstract

**Objectives:**

Aortic valve sparing-aortic root replacement (David procedure) has not been routinely performed *via* minimally invasive access due to its complexity. We compared our results of elective David procedure *via* minimally invasive access to those *via* a full sternotomy.

**Methods:**

Between 1993 and 2019, a total of 732 patients underwent a valve sparing root replacement (David) procedure. Out of these, 220 patients underwent elective David-I procedure (isolated) without any other concomitant procedures at our center. Patients were assigned to either group A (*n* = 42, mini-access) or group B (*n* = 178, full sternotomy).

**Results:**

Cardiopulmonary bypass time were 188.5 ± 35.4 min in group A and 149.0 (135.5–167.5) in group B (*p* < 0.001). Aortic cross-clamp time were 126.2 ± 27.2 min in group A and 110.0 (97.0–126.0) in group B (*p* < 0.001). Post-operative echocardiography showed aortic insufficiency ≤ I° in 41 (100%) patients of group A and 155 (95%) of group B. In-hospital mortality was 2.4% (*n* = 1) in group A and 0% (*n* = 0) in group B (*p* = 0.191). Perioperative stroke occurred in 1 (2.4%) patient of group A and 2 (1.1%) patients of group B (*p* = 0.483). Reexploration for bleeding was necessary in 4 (9.5%) patients of group A and 7 (3.9%) of group B (*p* = 0.232). Follow-up was complete for 98% of all patients. The 1-, 2-, 4-, and 6-year survival rates were: 97, 97, 97, and 97%, in group A (mini-access) and 99, 96, 95, and 92% in group B (full sternotomy), respectively. The rates for freedom from valve-related re-operation at 1, 2, 4, and 6 years after initial surgery were: 97, 95, 95, and 84% in group A and 97, 95, 91, and 90% in group B, respectively.

**Conclusion:**

Early post-operative results after David procedure *via* minimally invasive access are comparable to conventional full sternotomy. Meticulous attention to hemostasis is a critical factor during minimally access David procedures. Long-term outcome including the durability of the reimplanted aortic valve seems to be comparable, too.

## Introduction

Minimally invasive access cardiac surgery has gained broader clinical application due to potential benefits of reduced surgical trauma and pain (1). It has been reported that patients have less pain and recover quicker from surgery (2). Especially in the field of mitral valve surgery, minimal access surgery has evolved into the standard of care in many centers.

Aortic valve-sparing root reimplantation (AVSRR) was introduced by David (3) and has become an established procedure for the treatment of combined pathologies of the ascending aorta and the aortic valve (4, 5). However, due to its complexity, David procedure is still not performed routinely *via* minimally invasive access. We started to perform AVSRR *via* mini access in 2011 and have reported our initial experience in 2015 (6). The study focusing on our initial experience comprised the first 26 patients who underwent AVSRR *via* upper hemi-sternotomy. Since then, we have gained more experience with this approach. Only few other centers have reported their experience with AVSRR through an upper hemi-sternotomy (7–9).

The present study was designed to compare patients who undergo AVSRR with mini-sternotomy with those with conventional full sternotomy.

## Methods

### Ethics

This is a retrospective study with follow-up. This study has been approved by our institution's Ethics Committee (Nr. 3552-2017). Thus, this study was in line with our institution's ethical policies and standards.

### Study population

Our institution's database was screened for AVSRR (*n* = 732 patients) that have been performed between 1993 and 2019. All patients with concomitant procedures as well as emergent acute aortic dissection type A were excluded and only elective cases were included. Only patients who received isolated AVSRR were included. We identified 220 patients who matched these criteria. The patients were assigned to group A if access was established *via* a minimally invasive upper hemi-sternotomy (*n* = 42) or group B if access was achieved *via* a conventional full sternotomy (*n* = 178).

### Surgical technique

All patients in this study underwent AVSRR with a straight tube graft (David-I). Concomitant procedures were not performed. A detailed description of our center's surgical technique of AVSRR can be found in previous publications (10), and our technique of establishing minimally invasive access in AVSRR has been published before, too (6). In brief, we perform an upper partial hemi-sternotomy into the 3rd or 4th intercostal space to establish access.

### Post-operative follow up

We obtained individual consent from patients to allow for follow-up examination. Follow-up was performed as suggested by common guidelines (11). We contacted patients by telephone or met them in our center's aortic clinic. We contacted primary care physicians and cardiologists to obtain the most recent echocardiography results.

### Statistical analysis

The data analysis was performed by the usage of SPSS 26 Statistics software (IBM Corp. Released 2019. IBM SPSS Statistics for Windows, Version 26.0. Armonk, NY: IBM Corp.). Normal distribution of variables was analyzed with the Shapiro Wilk test. Normally distributed continuous variables are stated as mean ± standard deviation, while continuous variables without normal distribution are stated as median + interquartile range. Continuous variables were analyzed with the Mann Whitney *U*-test, while categorical variables were compared with the Fisher's exact test. Kaplan-Meier analysis was used for evaluation of both survival and re-operation of the aortic valve, and the log-rank test was used to test for differences. A value of *p* < 0.05 was considered statistically significant.

## Results

### Patient demographics

The patient characteristics are shown in Table 1. All patient demographics were distributed equally between the two groups, except for BMI and Marfan syndrome. The mean age of the entire group was 47.0 (34.0–61.0) years. The majority of patients (*n* = 147, 72.4%) had significant aortic insufficiency (grade ≥ II°). All cases underwent elective surgery.

**Table 1:** Patient demographics.

	**Entire group**	**Minimally invasive**	**Full sternotomy**	***P*-value**
Total patients (*n*)	*n* = 220	*n* = 42	*n* = 178	
Sex (male)	*n* = 162 (73.6%)	*n* = 35 (83.3%)	*n* = 127 (71.3%)	0.113
Age (years)	47.0 (34.0–61.0)	47.2 ± 14.2	47.5 (34.0–62.0)	0.990
BMI (kg/m^2^)	25.4 ± 4.8	27.5 ± 3.9	24.9 ± 4.9	0.002
Hypertension	111 (50.5%)	23 (54.8%)	88 (49.4%)	0.535
Diabetes	7 (3.2%)	2 (4.8%)	5 (2.8%)	0.621
COPD	5 (2.3%)	0 (0%)	6 (3.4%)	0.586
CAD	3 (1.4%)	1 (2.4%)	2 (1.1%)	0.472
Marfan syndrome	*n* = 62 (28.2%)	*n* = 5 (11.9 %)	*n* = 57 (32.0%)	0.009
Re-Do	*n* = 6 (2.7%)	*n* = 0 (0.0%)	*n* = 6 (3.4%)	0.598
Echocardiography	*n* = 203	*n* = 39	*n* = 164	
AI 0–1	*n* = 16 (7.9%)	*n* = 4 (10.2%)	*n* = 12 (7.3%)	
AI 1	*n* = 28 (13.8%)	*n* = 5 (12.8%)	*n* = 23 (14.0%)	
AI 1–2	*n* = 12 (5.9%)	*n* = 1 (2.6%)	*n* = 11 (6.7%)	
AI ≥ 2	*n* = 147 (72.4%)	*n* = 29 (74.4%)	*n* = 118 (72.0%)	

AI, aortic insufficiency; BMI, body mass index; COPD, chronic obstructive pulmonary disease; CAD, coronary artery disease.

### Intra-operative and early post-operative outcome

The intraoperative results are shown in Table 2. The cardiopulmonary bypass time was 188.5 ± 35.4 min in group A and 149.0 (135.5–167.5) in group B (*p* < 0.001). Aortic cross-clamp time was 126.2 ± 27.2 min in group A and 110.0 (97.0–126.0) in group B (*p* < 0.001). The post-operative outcome is shown in Table 3. The post-operative echocardiography was available for 205 (93%) patients and showed aortic insufficiency ≤ I° in 41 (100%) patients of group A and 155 (95%) of group B. Reexploration for bleeding was necessary in 4 (9.5%) patients of group A and 7 (3.9%) of group B (*p* = 0.232). Perioperative stroke occurred in 1 (2.4%) patient of group A and 2 (1.1%) patients of group B (*p* = 0.483). In-hospital mortality was 2.4% (*n* = 1) in group A and 0% (*n* = 0) in group B (*p* = 0.191). The patient who deceased underwent mini access AVSRR and died from multi-organ failure.

**Table 2:** Intraoperative data.

	**Entire group**	**Minimally invasive**	**Full sternotomy**	***P*-value**
Total patients (*n*)	*n* = 220	*n* = 42	*n* = 178	
Aortic x-clamp time (minutes)	113.0 (100.0–128.0)	126.2 ± 27.2	110.0 (97.0–126.0)	*p* < 0.001
CPB time (minutes)	156.0 (138.0–178.0)	188.5 ± 35.4	149.0 (135.5–167.5)	*p* < 0.001
PBC (units)	0 (0–2)	0 (0–1)	0 (0–2)	0.119
GFP (units)	0 (0–3)	0 (0–0)	0 (0–4)	< 0.001
Platelets (units)	0 (0–2)	0 (0–2)	0 (0–1)	0.339
Control echocardiography	*n* = 205	*n* = 41	*n* = 164	
AI 0–1	*n* = 143 (69.7%)	*n* = 34 (82.9%)	*n* = 109 (66.5%)	
AI 1	*n* = 53 (25.9%)	*n* = 7 (17.1%)	*n* = 46 (28.0)	
AI 1–2	*n* = 5 (2.4%)	*n* = 0 (0.0%)	*n* = 5 (3.1%)	
AI ≥ 2	*n* = 4 (2.0%)	*n* = 0 (0.0%)	*n* = 4 (2.4%)	

CPB, cardiopulmonary bypass; AI, aortic insufficiency.

The number of PBC, GFP and platelets given in this table refers to the intraoperatively administered products only.

**Table 3:** Post-operative outcome.

	**Entire group**	**Minimally invasive**	**Full sternotomy**	***P*-value**
Total patients (*n*)	*n* = 220	*n* = 42	*n* = 178	
Mech. ventilation time (days)	0.5 (0.3–0.6)	0.5 (0.3–0.6)	0.4 (0.3–0.6)	0.937
Tracheostomy	4 (1.8%)	0 (0%)	4 (2.2%)	1.000
ICU stay (days)	1.0 (1.0–2.0)	1.0 (1.0–2.3)	1.0 (1.0–2.0)	0.364
In-hospital mortality	*n* = 1 (0.5%)	*n* = 1 (2.4%)	*n* = 0 (0%)	0.191
PBC (units)	2 (0–4)	2 (0–4)	2 (0–4)	0.961
FFP (units)	3 (0–4)	0 (0–2)	3 (2–5)	< 0.001
Platelets (units)	0 (0–2)	1 (0–2)	0 (0–2)	0.149
Reexploration for bleeding	*n* = 11 (5.0%)	*n* = 4 (9.5%)	*n* = 7 (3.9%)	0.232
Stroke	*n* = 3 (1.4%)	*n* = 1 (2.4%)	*n* = 2 (1.1%)	0.483
Dialysis	*n* = 1 (0.5%)	*n* = 1 (2.4%)	*n* = 0 (0%)	0.196

ICU, intensive care unit; PBC, packed blood cells; FFP, fresh frozen plasma.

The number of PBC, GFP and platelets given in this table refers to the total units administered during the entire hospital stay.

### Long-term outcome

**Figure 1:**
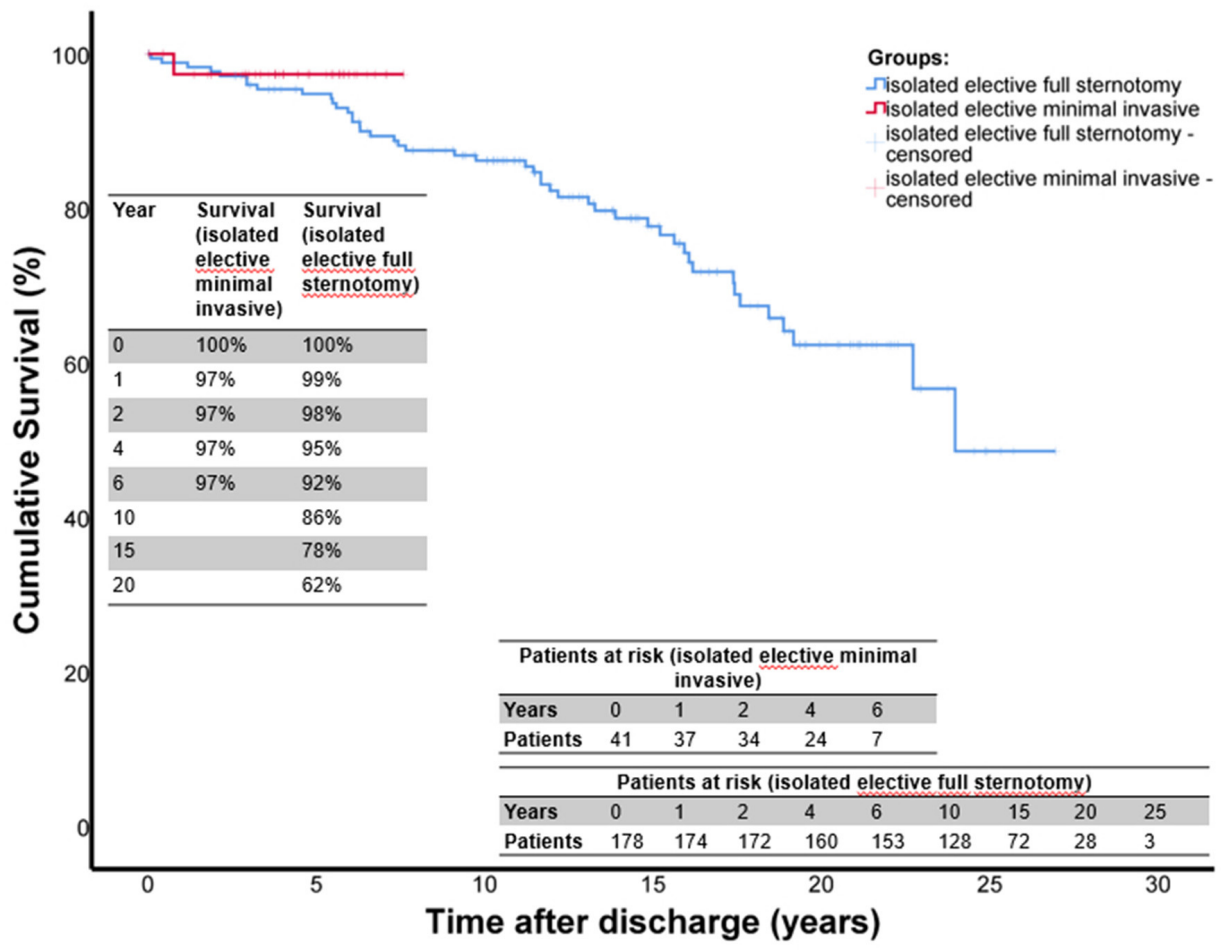
Survival after isolated elective AVSRR. This figure shows the Kaplan Meier survival curves for patients who underwent isolated, elective David-I procedure. The red curve shows the mini access patients (group A) and the blue curve shows the full sternotomy patients (group B). Time origin on x-axis denotes day of surgery.

**Figure 2:**
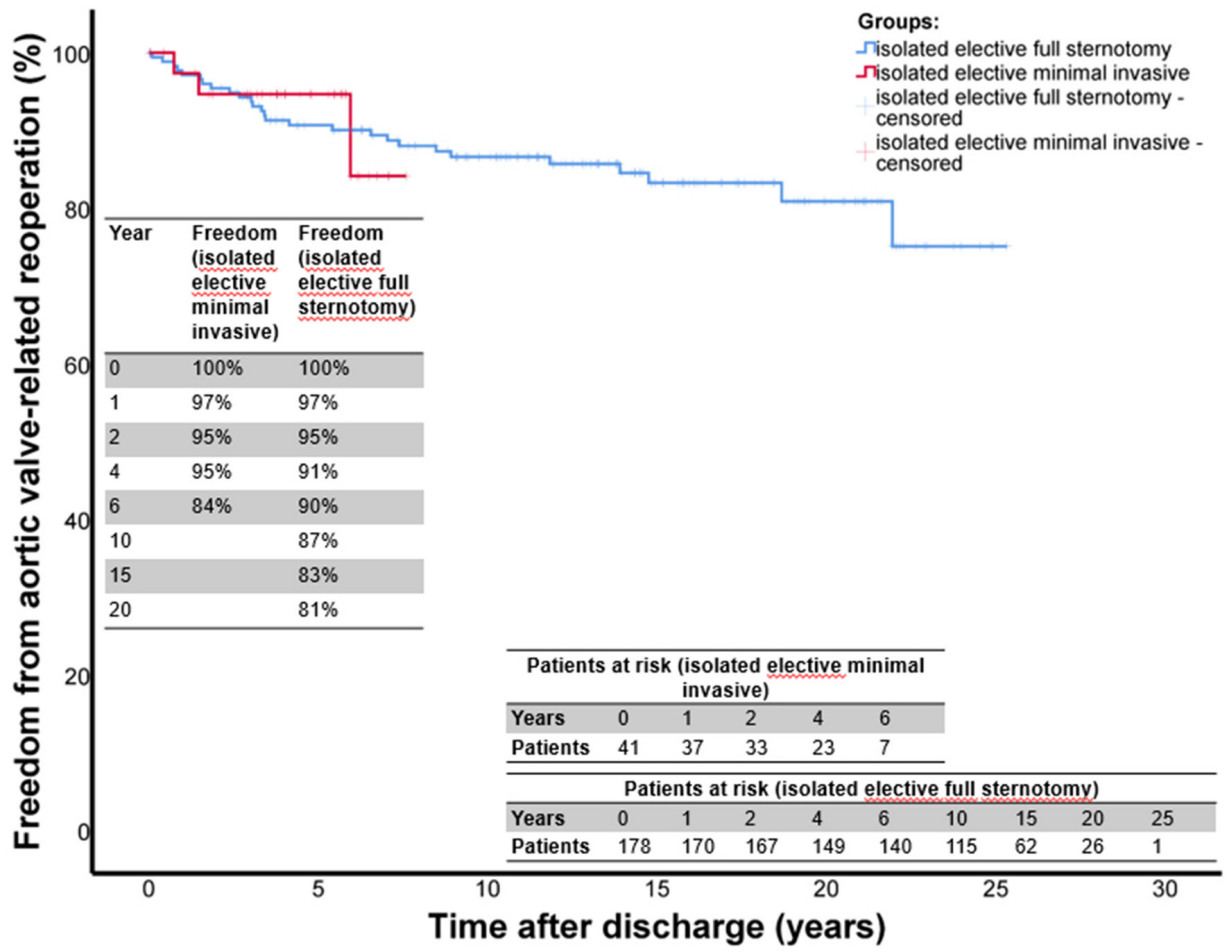
Freedom from aortic valve-related reoperation after isolated elective AVSRR. This figure shows the Kaplan Meier curves for freedom from aortic valve-related reoperation after isolated, elective David-I procedure. The red curve shows the mini access patients (group A) and the blue curve shows the full sternotomy patients (group B). Time origin on x-axis denotes day of surgery.

Follow-up was complete for 98% of all patients. The mean follow-up time was 11.5 ± 6.7 years for the entire group. The mean follow-up times for group A was 4.2 ± 2.1 years and 13.2 ± 6.2 years for group B, respectively. The long-term survival is shown in Figure 1. The 1-, 2-, 4-, and 6-year survival rates were: 97, 97, 97, and 97%, in group A (mini-access) and 99, 96, 95, and 92% in group B (full sternotomy), respectively. The freedom from aortic valve-related reoperation is shown in Figure 2. The rates for freedom from valve-related re-operation at 1, 2, 4, and 6 years after initial surgery were: 97, 95, 95, and 84% in group A and 97, 95, 91, and 90% in group B, respectively. There was a total of 31 patients who required aortic valve-associated reoperation. The reasons for reoperation were: aortic insufficiency in 22 patients, aortic stenosis in 5 patients, and endocarditis in 4 patients.

## Discussion

This study summarizes our center's experience with minimally invasive AVSRR (David-I) *via* upper hemi-sternotomy, and provides a direct comparison between mini-sternotomy and full sternotomy. The early post-operative results after David procedure *via* minimally invasive access are comparable to conventional full sternotomy. Meticulous attention to hemostasis is a critical factor during minimal access David procedures. Minimal access surgery for cosmetic and aesthetic reasons is an important factor for young patients. In elderly patients, the possibility of shorter convalescence period is the main advantage of minimal access surgery.

### General and technical considerations

When AVSRR was introduced in the early 1990s, we adopted this promising technique very early in 1993 at our center. Initially, all AVSRR procedures were performed *via* full sternotomy. With growing experience and expertise, we started performing AVSRR through minimally invasive access in 2013. Only surgeons with sufficient experience in AVSRR *via* full sternotomy perform this procedure through an upper hemi-sternotomy at our center. In the present study, a total of 4 surgeons performed minimal access AVSRR, while 18 surgeons performed David procedure *via* a full sternotomy.

With regards to selection criteria, we consider every patient eligible for AVSRR if it is an isolated David procedure if the surgeon has sufficient expertise. Concomitant hemiarch replacement can also be performed safe through an upper hemi-sternotomy. However, if there are any other concomitant cardiac surgical procedures (for instance coronary artery bypass grafting, total aortic arch replacement, or mitral valve surgery), a full sternotomy is performed.

When evaluating patients for mini-access AVSRR, we pay careful attention to the anatomic location of the aortic root on computed tomography scan. The scan determines whether the 3rd or 4th intercostal space is used for access.

Minimally invasive access AVSRR requires careful performance of the anastomoses, and meticulous hemostasis. It is key to achieve perfect hemostasis, because bleeding from the aortic root is hard to control in minimally invasive access cardiac surgery. Significant bleeding may even require another pump run.

### Early outcome

In the present study, both the cardiopulmonary bypass and the aortic cross clamp times were longer in the minimally invasive access group than in the full sternotomy group. However, this did not lead to an increased incidence of myocardial ischemia-related complications. We did not observe an increased rate for post-operative low cardiac output syndrome in the mini access group.

The usage of fresh frozen plasma during the operation and during the entire hospital course was significantly higher in the full sternotomy group. This can be explained by the more invasive access and trauma in the full sternotomy course. Further, this finding also underlines that meticulous hemostasis is very important during minimal access AVSRR.

There was only one perioperative death in the entire study, resulting in an overall in-hospital mortality of 0.5%. In comparison, the operative mortality in Tirone David's group was 1% (4). The patient who deceased in our study died because of multiorgan failure. This patient underwent mini access AVSRR, and since the mini access group is relatively small the in-hospital mortality is 2.4%. Although there was no early death in the full sternotomy group, we do not think that mini access was linked to the death of this patient. Given the low in-hospital mortality of 0.5% of the entire cohort, we think that this demonstrates that full aortic root replacement using a valve-sparing technique can be done extremely safe. Clearly, careful patient selection is important.

The perioperative incidence for permanent neurological deficit was 1.5% in the entire cohort. This is an encouraging low number, too. However, the rate for reexploration for bleeding was slightly higher in group A (mini access) than in group B (full sternotomy). Although minimally invasive cardiac surgery is known to reduce trauma and facilitate post-operative recovery (2), one has to assume that hemostasis in mini access aortic root surgery is more complicated. We conclude that more attention should be directed toward meticulous hemostasis in order to prevent reexploration.

The post-operative echocardiographic data showed comparable results in the two groups. For instance, echo showed aortic insufficiency ≤ I° in 41 (100%) patients of group A and 155 (95%) of group B. Therefore, we conclude that mini access does not compromise the quality of the preserved and reimplanted aortic valve.

### Long-term outcome

We started AVSRR in 1993 at our center and by now, we have done more than 700 AVSRR operations. Using a minimally invasive approach *via* upper partial sternotomy has been introduced later. First, mini access was applied to relatively simple operations such as aortic valve replacement, and later—with growing experience—also to more complicated procedures. We applied min access to AVSRR in 2013, almost 20 years after the first David procedure at our hospital. This explains the smaller sample size and the shorter follow up time of the mini access group when compared to the conventional group. In turn, it is difficult to compare and comment on the long-term durability and performance of the reimplanted aortic valve in group A. At least for the mid-term outcome, we observed no major difference in aortic valve-related reoperations between the two groups. The same seems to be true for mid-term survival. Future studies will have to clarify whether survival and aortic valve durability after mini access AVSRR are adequate in the long term.

Although we expect comparable long-term outcome after minimally invasive access AVSRR, we want to emphasize that only experienced surgeons should perform David procedure *via* mini access. Despite the encouraging outcome in the present study, David procedure remains a complex operation. Surgeons go through a learning phase until having sufficient results with this technique (12). Therefore, we think that a step-by-step approach is recommended to establish minimally invasive David procedure. Surgeons should have sufficient expertise and experience with AVSRR *via* full sternotomy before starting mini access. Then, surgeons should start with simple operations first through an upper hemi-sternotomy, such as conventional aortic valve replacement. With growing experience with this approach, more complicated procedures can be performed *via* mini access.

### Limitations

This is a retrospective study which carries all potential risks and disadvantages of this study type. The sample size of the mini access group is relatively small, and follow up time shorter than in the full sternotomy group. There is potential selection bias, as more experienced surgeons may have performed minimally invasive access cardiac surgery.

## Conclusions

The present study provides a direct comparison of AVSRR with a mini-sternotomy and conventional full sternotomy. The early post-operative results after David procedure *via* mini access are comparable to full sternotomy. Meticulous attention to hemostasis is a critical factor during minimally invasive access David procedures. Long-term outcome including the durability of the reimplanted aortic valve seems to be comparable, too, but longer follow up times are needed.

## Data availability statement

The datasets presented in this article are not readily available because the data underlying this article cannot be shared publicly because due to privacy issues. The International Committee of Medical Journal Editors (ICMJE) emphasizes that patients and study participants have a right to privacy that should not be infringed without informed consent. Study participants should know exactly how their data will be used and shared. Although our patients gave informed consent to participate in this study, we did not ask them to give consent to share their anonymized data publicly. For this reason and to be in line with the recommendations of the ICMJE, we cannot make the data publicly available. Reasonable requests to the corresponding author will be evaluated. Requests to access the datasets should be directed to shrestha.malakh.lal@mh-hannover.de.

## Ethics statement

The studies involving human participants were reviewed and approved by Hannover Medical School Ethics Committee. The patients/participants provided their written informed consent to participate in this study.

## Author contributions

All authors were involved in the conceptualization, data collection, data analysis, and writing the article.

## Funding

This study was funded by Departmental Grants.

## Conflict of interest

The authors declare that the research was conducted in the absence of any commercial or financial relationships that could be construed as a potential conflict of interest.

## Publisher's note

All claims expressed in this article are solely those of the authors and do not necessarily represent those of their affiliated organizations, or those of the publisher, the editors and the reviewers. Any product that may be evaluated in this article, or claim that may be made by its manufacturer, is not guaranteed or endorsed by the publisher.
